# Characterization and Prebiotic Effect of the Resistant Starch from Purple Sweet Potato

**DOI:** 10.3390/molecules21070932

**Published:** 2016-07-19

**Authors:** Yafeng Zheng, Qi Wang, Baoyu Li, Liangmei Lin, Rosa Tundis, Monica R. Loizzo, Baodong Zheng, Jianbo Xiao

**Affiliations:** 1 College of Food Science, Fujian Agriculture and Forestry University, Fuzhou 350002, China; zyffst@163.com (Y.Z.); nkywq@163.com (Q.W.); Libaoyuyjy@163.com (B.L.); liulingpiaoshi@163.com (L.L.); zbdfst@163.com (B.Z.); 2 Fujian Provincial Key Laboratory of Quality Science and Processing Technology in Special Starch, Fuzhou 350002, China; 3 Institute of Agricultural Engineering, Fujian Academy of Agriculture Sciences, Fuzhou 350003, China; 4 Department of Pharmacy, Health and Nutritional Sciences, University of Calabria, Via P. Bucci, 87036 Rende (CS), Italy; rosa.tundis@unical.it (R.T.); monica_rosa.loizzo@unical.it (M.R.L.)

**Keywords:** purple sweet potato, resistant starch, physicochemical properties, structure properties, bifidobacteria proliferation

## Abstract

Purple sweet potato starch is a potential resource for resistant starch production. The effects of heat-moisture treatment (HMT) and enzyme debranching combined heat-moisture treatment (EHMT) on the morphological, crystallinity and thermal properties of PSP starches were investigated. The results indicated that, after HMT or EHMT treatments, native starch granules with smooth surface was destroyed to form a more compact, irregular and sheet-like structure. The crystalline pattern was transformed from C-type to B-type with decreasing relative crystallinity. Due to stronger crystallites formed in modified starches, the swelling power and solubility of HMT and EHMT starch were decreased, while the transition temperatures and gelatinization enthalpy were significantly increased. In addition, HMT and EHMT exhibited greater effects on the proliferation of bifidobacteria compared with either glucose or high amylose maize starch.

## 1. Introduction

Sweet potato (*Ipomoea batatas* L.) originated from Latin America, and is now cultivated worldwide, especially in Africa and Asia. China accounts for most of worldwide sweet potato production. The skin and flesh colors of sweet potato vary from white to yellow-orange and deep purple. Purple sweet potatoes contain a significant content of anthocyanins [1], which are attractive natural colorants exhibiting strong free radical scavenging activity [2].

Purple sweet potato is a promising source of anthocyanins for the food industry, however, a large amount of starch is wasted as by-products. Sweet potato starch is suitable for the production of resistant starch [3,4], which is a value added starch product with increasing market demand.

The resistant starch is the starch fraction which escapes digestion in the small intestine of healthy individuals, but is completely or partially fermented in the colon [5]. Resistant starch has been proved to exhibit potential health benefits, including reducing the glycemic response [6], lowering blood cholesterol [7], acting as a functional probiotic [8], and increasing the production of short chain fatty acids in the large intestine [9].

Recently, various methods for the production of resistant starches from different resources have been reported, including heat-moisture treatment (HMT) [10], enzyme debranching [11], chemical modification [12], and cross-linking [13]. Among these methods, the heat-moisture treatment and enzyme debranching are safe and cost-effective methods for the production of resistant starches.

Although the physicochemical characteristics of native and resistant starches from sweet potato have been extensively studied [4,14,15], a small number of works has been reported on the structure and properties of resistant starch from purple sweet potato and its probiotic effect.

In the present study, resistant purple sweet potato starches were produced by HMT and enzyme debranching combined heat-moisture treatment (EHMT). The physicochemical and crystallinity properties of native, HMT and EHMT starches from purple sweet potato were characterized by scanning electron microscopy (SEM), X-ray diffraction (XRD), and Fourier Transform infrared (FT-IR) spectroscopy. In addition, the effects of these starches on the proliferation of bifidobacteria were assessed and compared with those of glucose (GLU) and high amylose maize starch (HAMS). The physicochemical properties of resistant starches from purple sweet potato prepared by HMT and EHMT were assessed in order to evidence the potential use as probiotics.

## 2. Results and Discussion

### 2.1. Chemical Composition

The chemical compositions of native, HMT and EHMT starches are shown in Table 1. The protein and lipid contents of native starch were quite low, indicating its high purity. The increase of protein content in EHMS might due to the residual Pullulanase enzyme after enzyme treatment. The amylose content in native starch was 29.91%, which was higher the values (13.33%–26.83%) recently reported for various sweet potato starches [16]. Considering that amylose content has a great effect on RS formation, purple sweet potato could be a potential resource for sweet potato RS production. The RS content of EHMT (17.16%) was significantly higher than HMT (14.23%), which would be attributed by the effect of Pullulanase enzyme on debranching of α-(1→6) linkage of amylopectin which is converted into small chain linear polysaccharides like amylase molecules [17]. Therefore, it also explained the increase of amylose content in EHMT (36.32%) compared to HMT (32.83%).

### 2.2. Morphological Characteristics

**Figure 1:**
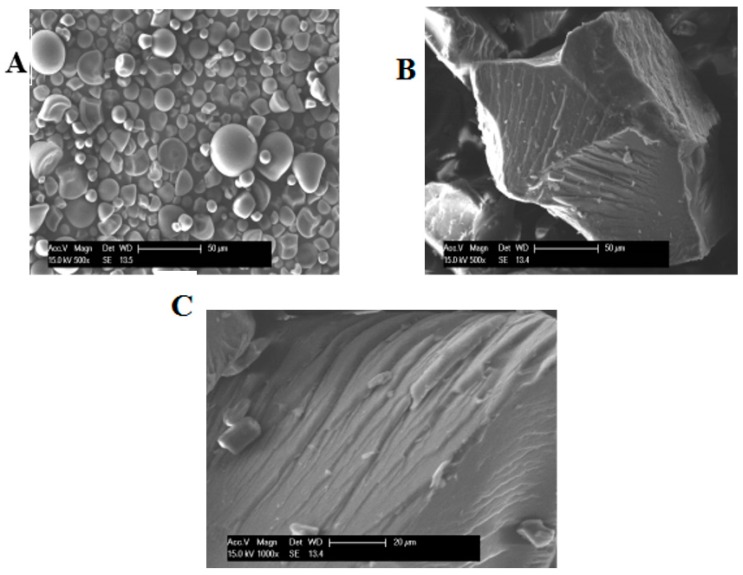
Scanning electronic micrographs of native, HMT and EHMT starches: (**A**) Native starch (×500); (**B**) HMT starch (×500); (**C**) EHMT starch (×1000).

The shapes and surface characteristics of native, HMT and EHMT starches prepared from purple sweet potatoes are shown in Figure 1. Native starch granules were polygonal, round, and bell shaped with smooth surfaces, which is in agreement with previous reports on sweet potato starch granules [16]. However, after treatment with the different methods, the granular shapes greatly changed. The structure of HMT starch was completely destroyed to form a compact, irregular and sheet-like structure. These observations are consistent with those reported for lotus seed starch [18] and red kidney beans starch [17]. The surface of HMT starch revealed the presence of layered strips, while gully shapes were observed on the surface of EHMT starch, whose structure is more compact. This observation could be attributed to the retrogradation of amylose chains, which resulted in a reorganization of the starch structure into a helical complex, stabilized by hydrogen bonds. Moreover, the density of the crystalline structure thus improves its resistance to enzyme digestion.

### 2.3. Swelling Power and Solubility

When starch is heated in water above its gelatinization temperature, the resulting irreversible changes result in loss of crystallinity, granule swelling and leakage of carbohydrate material [19]. Variation in the swelling power and solubility of the starches under different treatments could be caused by the variations in the associative bonding forces within the starch granules. Swelling power and solubility of the starch samples are presented in Table 2.

Compared to the swelling power of native starch (26.21 g/g), the swelling power of HMT starch decreased to 20.72 g/g, while EHMT starch exhibited the lowest swelling power of 17.21 g/g. During HMT, the internal of starch granules was rearranged, and the mobility of the molecules would be increased, and thus cause enhanced associations between interacting amylose-amylose and amylose-amylopectin chains. The resulting rigid structure could restrict the swelling of starch granules. This result was consistent with previous reports, where the swelling power was reduced during HMT of rice starch [20], sweet potato starch [3] and sorghum starch [21]. Debranching treatment by pullulanase produces partially debranched amylopectin which can act like amylase and create highly crystalline structures, and thus further reduce the swelling power of EHMT starch [11].

The solubility of native starch determined at 90 °C was 9.46%, which was relatively low compared to the sweet potato starches (8.56% to 19.97%) [16]. This might be caused by its small granule size and higher amylose content compared to other sweet potato cultivars [22]. After treatments, the solubility values of HMT and EHMT starch samples were decreased from 9.46% to 8.74% and 7.41%, respectively. Enhanced interaction between amylose-amylose and amylose-amylopectin chains, which leads to a decrease in the leaching of carbohydrate material was reported [23]. When debranching treatment by pullulanase was combined with HMT, the extent of interaction between starch chains was increased, which explained the lower solubility of EHMT starch.

### 2.4. Thermal Properties

The gelatinization transition temperatures (*T_o_*, *T_p_* and *T_c_*), the gelatinization temperature range (*T_c_*–*T_o_*) and gelatinization enthalpy (Δ*H*) of native, HMT and EHMT starch samples are presented in Table 2. According to the results, the thermal properties of starch samples were significantly changed during enzyme debranching and/or heat-moisture treatment.

Compared to the native starch, HMT has been shown to increase the gelatinization temperatures (most pronounced for *T_o_* and least for *T_c_*) and decrease the gelatinization temperature range, which is consistent to other starches [3,24]. It is reported that recrystallized amylopectin melts in the temperature range from 40 to 100 °C, while amylose crystallites melt at much higher temperature ranged from 120 to 170 °C [14]. As presented in Table 2, the transition temperatures of HMT starch were significantly increased, which could be attributed to the increased amylose crystallites content and structural stability during HMT. During debranching accompanying heat-moisture treatment, the amylose content in granules was increased as shown in Table 1, and stronger crystallites were formed to resistant the gelatinization, which caused higher gelatinization temperatures [25].

Gelatinization enthalpy (Δ*H*) represents an overall measure of crystallinity (quality and quantity) and is closely related to the loss of molecular order within the granule. Furthermore, the starch samples with higher RS content, which contributes to the increase of the perfection of double helical order and formation of stronger matrix or network, had a higher Δ*H* value. Therefore, as presented in Table 1 and Table 2, the starch samples with the higher RS content had the higher Δ*H* value.

### 2.5. X-ray Diffraction and Relative Crystallinity

According to the position of the X-ray diffraction peaks, the crystalline nature could be defined as A, B and C type. Reported by Cheetham and Tao [26], a typical A-type starch has strong reflections at a 2θ angle of about 15° and 23°, and an unresolved doublet at around 17° and 18°, while, a typical B-type starch has a strong peak at around 17°, a characteristic peak at about 5.6°, and some weak peaks at around 15°, 20°, 22°, and 24°. C-type starch is a mixture of both A and B-type. Generally, cereal starches generate A-type diffraction patterns; potato starches generate B-type patterns; and legume starch show C-type patterns [27]. However, sweet potato starch from different varieties have variable X-ray patterns [14,15,28].

**Figure 2:**
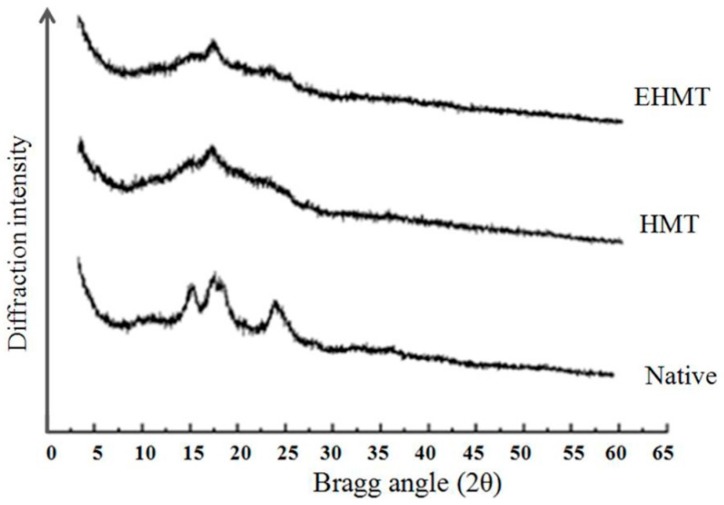
X-ray diffraction patterns of native, HMT and EHMT starches.

Figure 2 presents the X-ray diffraction patterns and relative crystallinity of native, HMT and EHMT purple sweet starch samples. Native purple sweet potato starch showed an unresolved doublet at 17.1° and 18.1°, a weak intensity at angle 2θ values of 27.4°, and two relatively stronger reflection intensities at 2θ angle values of 15.5° and 24.8°. The results indicated that its crystalline pattern lied between the A- and B- type pattern, which should be classed as C-type with the relative crystallinity of 36.14%. After heat-moisture treatment, a significant change happened to the X-ray diffraction patterns of HMT starch samples with reflection intensities at 2θ angle values of 15.5°, 17.1°, 24.8° and 27.4°, and the relative crystallinity was decreased to 33.72%. The doublet at 17.1° and 18.1° merged into a strong peak at 17.1°, and the strong peaks at 15.5° and 24.8° were converted into shoulders, indicating the transformation from C-type to B-type. Similar results were also found for the resistant starches prepared from red kidney beans starch [17] and lotus seed starch [29].The transformation of crystalline pattern of HMT starch sample could be attributed to the retrogradation at low temperature, which caused increased amylose content and the formation of more ordered double helices within the crystalline domains during HMT. The same change was found in the EHMT starch sample, except for a slight stronger intensity at 2θ angle values of 17.1°.

Compared with the relative crystallinity of native starch sample (36.14%), those of HMT and EHMT starches (33.72% and 35.68%) were both decreased, which was due to the damage of the crystalline region and partial gelatinization of the starch granules during HMT. The enzyme debranching combined HMT caused a higher crystallinity than HMT alone, which could be explained by the higher RS content after debranching [3].

### 2.6. FT-IR Spectroscopy

Infrared spectroscopy provides information about bonds whose dipole moment changes during vibration, which enables analysis of the short-order range such as chain conformation and double helical order of starch granules. The changes in peak intensity indicate characteristics of the conformational change in the starch.

**Figure 3:**
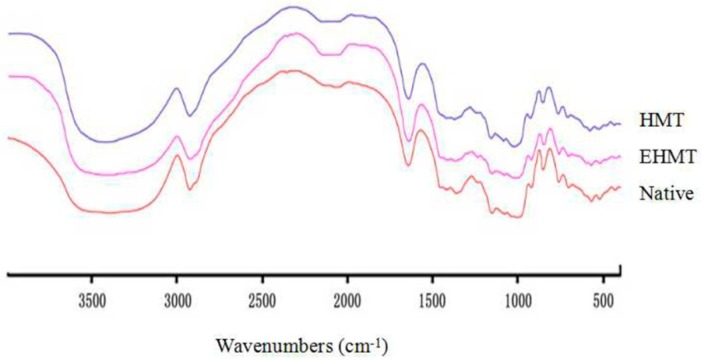
Infrared spectra of native, HMT and EHMT starches.

Figure 3 shows the FT-IR spectra of native, HMT and EHMT starches from purple sweet potato. Both native and treated starch samples showed similar FT-IR spectra results, indicating no changes in chemical groups. Therefore, the production of HMT and EHMT starches has resulted in physical modifications. According to the literatures [30,31,32], the peak around 3420 cm^−1^ was assigned to hydroxyl group stretching vibrations, and the broad band was due to the complex vibration stretches of intermolecular hydroxyl groups; the peak at around 2930 and 1640 cm^−1^ were associated to −CH_2_ stretching vibrations and deformation vibrations of the hydroxyl groups in water, respectively. The weak bands at 1370 and 1420 cm^−1^ were attributed to the twisting and bending shake of −CH_2_.

The absorption region from 800 to 1200 cm^−1^ reflects C-C, C-O, C-H stretching vibration and COH bending modes [33], changes in starch polymer conformation, and the process of hydration [34]. The bands at around 1159 cm^−1^ and 1083 cm^−1^ are associated to the ordered structures of starch, while the band at 991 cm^−1^ is associated to the amorphous structures of starch [35]. The amount of short-range ordering of the starch samples are related linearly to the ratios of R (1158/991 cm^−1^) [36]. The ratios of R (1158/991 cm^−1^) in native, HMT and EHMT starches were 0.725, 0.755 and 0.759. The greater R values of HMT and EHMT would indicate the higher levels of order in the external regions of the starch granule.

### 2.7. Probiotic Effects

**Figure 4:**
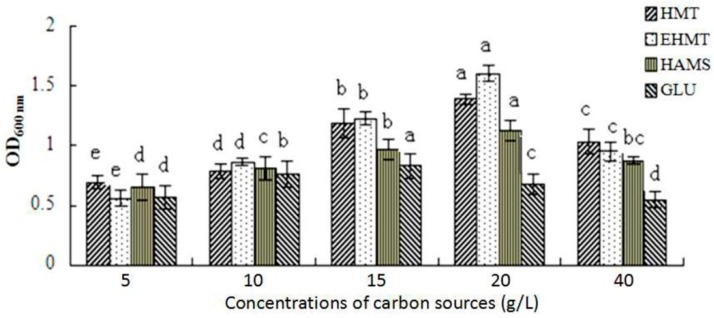
Effect of different carbon source concentrations on the number of bifidobacteria.

To evaluate the potential probiotic effects of HMT and EHMT starch from purple sweet potato, different carbon sources including glucose (GLU, negative control), high amylose maize starch (HAMS, positive control), HMT starch and EHMT starch were applied to the media of bifidobacteria culture. Figure 4 shows the proliferation of bifidobacteria in the media using different carbon sources. After 50 h of fermentation, with a carbon source concentration in the range 5–20 g/L, the number of bifidobacteria (expressed as the OD_600 nm_ value) increased with the higher concentration of the different carbon sources, and reached the highest level at the concentration of 20 g/L. The GLU group reached the highest level at the concentration of 15 g/L.

At a carbon source concentration of 40 g/L, the number of bifidobacteria decreased in all the tested media. These data might be attributed to a high osmotic pressure induced by high concentrations of carbon sources [18]. Compared with the media containing HAMS, which has been proved to be a promising probiotic [37], both types of RS from purple sweet potato, especially EHMT starch, showed better prebiotic effects on bifidobacteria on the different carbon source concentrations. The effect may be attributed to its stable double helix structure and rough surfaces.

**Figure 5:**
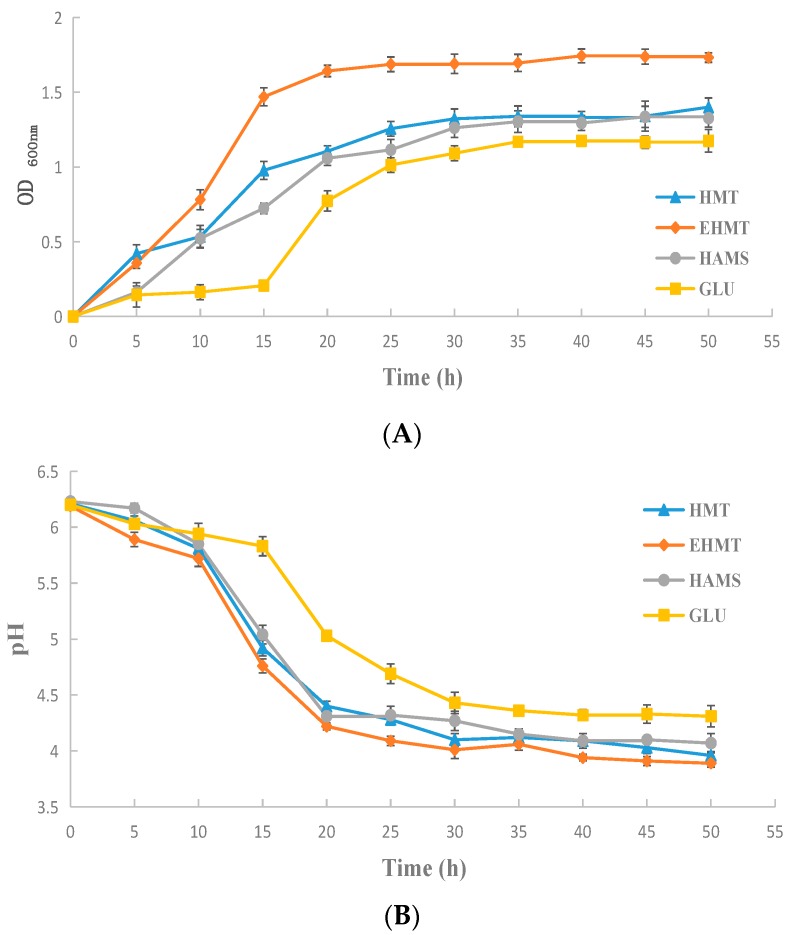
(**A**) Growth curves of bifidobacteria incubated in media with 20 g/L of GLU, HMT, EHMT, or HAMS; (**B**) Variation trend of pH values of the media during cultivation.

To further assess the probiotic effects, the growth curves of bifidobacteria and the variation trend of pH values of media during cultivation were plotted during cultivation. Based on the previous result (Figure 4), the carbon concentration in the media was 20 g/L. According to Figure 5A, the corresponding OD_600 nm_ values of media containing HAMS, HMT or EHMT starch were significantly higher than that of media containing GLU during cultivation, while EHMT starch exhibited an obviously higher value than HAMS and HMT starch. In the medium, the increase of bifidobacteria accompanied a reduction in pH during fermentation, which is due to the accumulated metabolites. As shown in Figure 5B, the rate of decrease of the pH value of the media was identical to the increase rate of bifidobacteria, and pH values of the media were lowered to 3.89–4.31 and the bifidobacteria growth remained stable at the end of fermentation. These results indicated the great potentiality of using HMT and EHMT starch from purple sweet potato as probiotics in the food industry.

## 3. Material and Methods

### 3.1. Preparation of Purple Sweet Potato Starch

The purple sweet potato Xinyin No.1, a popular cultivar in south China, was freshly harvested, and the tubers were washed, peeled and cut into small pieces, and then blended with three times as much water (by weight) in a domestic blender for 3–4 min. The resulting slurry was passed through fine gauze to separate the cell debris and the suspension was blended with water and filtered three more times to wash the starch into the filtrate, and allowed to settle overnight at room temperature. The precipitated fraction was collected and dried in a DJG-9053A oven (Yiheng Instrument Co., Ltd., Shanghai, China) at 45 °C for 24 h, and then ground using a FW135 grinder (Taisite Instrument Co., Ltd., Tianjin, China), and filtered using 100-mesh sieve to obtain the purple sweet potato starch [16]. The resulting starch was packaged in tightly covered polypropylene containers and stored in a refrigerator at 4 °C prior to use.

### 3.2. Preparation of Purple Sweet Potato Resistant Starch

#### 3.2.1. Preparation of Heat-Moisture-Treated (HMT) Starch

Purple sweet potato starch (100 g, db) was mixed with distilled water (250 mL), and moisture content was adjusted to 35%. The starch slurry was pre-gelatinized in a boiling water bath (110 °C for 40 min). The sample was cooled to room temperature and stored at 4 °C overnight in a refrigerator. Successively, the sample was dried at 60 °C for 16 h and milled to pass a 100-mesh sieve for further analyses.

#### 3.2.2. Preparation of Enzyme Debranching Combined Heat-Moisture-Treated Starch (EHMT)

Purple sweet potato starch (100g, 35% *w*/*v*) was gelatinized with distilled water by stirring. The starch slurry was pre-gelatinized in a boiling water bath, and then cooled to 60 °C, and debranched by pullulanase (1000 ASPU/g, obtained from Genencor China Bio-Products Co., Ltd., Wuxi, China) at the concentration of 25 ASPU/g with a pH of 4.8 [28]. After the reaction for 12 h, enzyme deactivation was carried out at 95 °C for 15 min, and then pH value was adjusted to 6.0.

The sample was heated at 110 °C for 40 min, and then cooled to room temperature and stored at 4 °C overnight in a refrigerator. The resulting starch was dried at 60 °C for 16 h and ground to pass a 100-mesh sieve for further analyses.

### 3.3. Resistant Starch Determination

The resistant starch content was determined with Resistant Starch Assay kit (Megazyme, Bray, Ireland) with the description of Association of Official Analytical Chemists (AOAC) 2002.02.

### 3.4. Scanning Electron Microscopy

The dried starch samples were coated with a thin film of gold (10 nm) in vacuum situations and the morphology of starch samples were observed under a scanning electron microscopy (PHILIPS-XL30, Eindhoven, The Netherlands).

### 3.5. Chemical Analysis

Moisture, lipid, and protein contents of starch samples were determined according to the methods described by Miao and co-workers [38]. Total amylose content was determined using DPCZ-II amylase content analyzer (Onisen Technology Co., Ltd., Shenzhen, China) according to the manufacturer’s instructions.

### 3.6. Water Solubility and Swelling Power

Water solubility and swelling power were determined on the basis of the method described by a previous report with slight modifications [39].The starch sample (0.6 g, db) was suspended in 30 mL of distilled water, and heated in a water bath at 90 °C for 30 min with constant mixing. The resulting slurries were cooled to room temperature in cold water. After centrifuging at 2000× *g* for 20 min, the supernatant was placed in an oven at 105 °C for 4 h to obtain the weight of dissolved starch, and the sediment of the centrifugation was also weighted. The solubility (S, %) and swelling power (SP, g/g,) was calculated as follows:

S = mass of dried supernatant/mass of dry starch × 100%(1)

SP = sediment weight/mass of dry starch × (1 − S)(2)

### 3.7. Thermal Analysis

Thermal properties were analyzed using an F3 200 Maria Differential Scanning Calorimeter (Netzsch, Selb, Germany) according to the reported method with slight modifications [17]. A starch sample (3 mg, db) was weighed into an aluminum pan and mixed with 14 µL of distilled water. The pans were hermetically sealed and equilibrated at room temperature for 24 h. Using an empty pan as the reference, the samples were scanned from 30 °C to 120 °C with at heating rate 10 °C/min and the nitrogen flow rate of 30 mL/min.

### 3.8. X-ray Diffraction and Relative Crystallinity

The starch samples were submitted to an X-ray diffractometer (X’Pert Pro MPD, Philips, Almelo, The Netherlands) operating at 40 KV and 30 mA at diffraction angle 2θ of 5° to 45° at speed of 2°/min. The graphs were analyzed by PeakFit 4.12, and then the relative crystallinity was calculated.

### 3.9. Fourier Transform Infrared Spectroscopy (FT-IR)

The IR spectrum of starch was recorded on a Nicolet Avatar 360 FR-IR spectrometer (Thermo Fisher Scientific, Waltham, MA, USA) at room temperature. The starch sample (2 mg) was blended with KBr powder (150 mg) by grinding for 10 min under the infrared lamp, and pressed into tablets before measurement. A region from 400 to 4000 cm^−1^ was used for scanning at a resolution of 4 cm^−1^ for 32 scans.

### 3.10. Proliferation Rate of Bifidobacteria

To investigate and compare the prebiotic effects, four types of carbohydrates were used as carbon source in the mediums. The fermentation mediums containing glucose (GLU; Sinopharm Chemical Reagent Co., Ltd., Shanghai, China), HMT, EHMT, or HAMS (60% amylose, Best-starch Creatmaterial Co., Ltd., Fujian, China) were prepared with the various carbon source concentration of 5, 10, 15, 20 or 40 g/L. The resulting fermentation mediums were sterilized at 115 °C for 20 min.

To get an inoculum, bifidobacteria (Livzon Pharmaceutical Group Inc., Guangdong, China) were incubated for 48 h in 15 mL of fermentation medium with glucose in anaerobic bags (Mitsubishi Gas Chemical, Tokyo, Japan). The resulting inoculum (2 mL, 16%, *v*/*v*) was transferred to 15 mL of fermentation medium. The incubation of bifidobacteria was performed in an anaerobic incubator (W-zipper Standing-Pouch, Mitsubishi Gas Chemical) at 37 °C. The proliferation rate of bifidobacteria was determined by plotting the OD_600 nm_ and pH values of the fermentation mediums at 0, 5, 10, 15, 20, 25, 30, 35, 40, 45 and 50 h, respectively [8,18]. Fermentation experiments were conducted in triplicate.

### 3.11. Statistical Analysis

All data was analyzed by DPS 9.05 system (Science Press, Beijing, China) or Peakfit 4.12 software (SeaSolve software Inc., Framingham, MA, USA). Statistical significance was set to *p* < 0.05.

## 4. Conclusions

Native PSP starch is a cheap and abundant starch containing relatively high amylose content, which makes it a potential resource for RS production. After heat-moisture treatment (HMT) or enzyme debranching combined heat-moisture treatment (EHMT), the morphological, crystallinity and thermal properties of PSP starches were significantly modified. The native starch granules structure was destroyed to form a more compact, irregular and sheet-like structure. The crystalline pattern was transformed from C-type to B-type with decreasing relative crystallinity. The swelling power and solubility of HMT and EHMT starch were decreased, due to the enhanced interactions between amylose-amylose and amylose-amylopectin chains. Stronger crystallites were formed in modified starches, and thus the transition temperatures and gelatinization enthalpy were significantly increased. These gelatinization properties indicate that HMT and EHMT starches could be stable during high temperature processing. In addition, HMT and EHMT exhibited greater effects on the proliferation of bifidobacteria compared with growth in the presence of HAMS, indicating the great potentiality of using them as probiotics in food industry.

## Figures and Tables

**Table 1:** The chemical composition of native, HMT and EHMT starch samples *.

Samples	Moisture (%)	Protein (%)	Lipid (%)	Amylose (%)	RS (%)
Native	5.03 ± 0.19 ^a^	0.41 ± 0.01 ^a^	0.11 ± 0.01 ^a^	29.91 ± 0.14 ^a^	5.02 ± 0.21 ^a^
HMT	4.92 ± 0.08 ^a^	0.38 ± 0.04 ^a^	0.17 ± 0.01 ^b^	32.83 ± 0.13 ^b^	14.23 ± 0.55 ^b^
EHMT	4.67 ± 0.07 ^b^	1.67 ± 0.07 ^b^	0.19 ± 0.02 ^c^	36.62 ± 0.25 ^c^	17.16 ± 0.63 ^c^

* Results are expressed as mean ± standard deviation; means with different superscripts (a, b, c) in the same column are significantly different (*p* < 0.05).

**Table 2:** Swelling power, solubility and thermal properties of native, HMT and EHMT starches.

Samples	SP (g/g)	S (%)	*T_o_* (°C)	*T_p_* (°C)	*T_c_* (°C)	*T_c_*–*T_o_* (°C)	Δ*H* (J/g)
Native	26.21	9.46	67.2	80.6	87.8	20.6	21.26
HMT	20.72	8.74	106.7	115.2	124.1	17.4	25.53
EHMT	17.21	7.41	135.9	143.3	152.8	16.9	26.78

SP, swelling power; S, solubility; *T_o_*, onset temperature; *T_p_*, peak temperature; *T_c_*, conclusion temperature; *T_c_*–*T_o_*, gelatinization temperature range; Δ*H*, gelatinization enthalpy.

## References

[1] Truong V.-D., Deighton N., Thompson R.T., McFeeters R.F., Dean L.O., Pecota K.V., Yencho G.C. (2010). Characterization of Anthocyanins and Anthocyanidins in Purple-Fleshed Sweetpotatoes by HPLC-DAD/ESI-MS/MS. J. Agric. Food Chem..

[2] Kano N., Takayanagi T., Harada K., Makino K., Ishikawa F. (2005). Antioxidative activity of anthocyanins from purple sweet potato, Ipomoera batatas cultivar Ayamurasaki. Biosci. Biotechnol. Biochem..

[3] Huang T.-T., Zhou D.-N., Jin Z.-Y., Xu X.-M., Chen H.-Q. (2016). Effect of repeated heat-moisture treatments on digestibility, physicochemical and structural properties of sweet potato starch. Food Hydrocoll..

[4] Xia H., Li Y., Gao Q. (2016). Preparation and properties of RS4 citrate sweet potato starch by heat-moisture treatment. Food Hydrocoll..

[5] Englyst H.N., Kingman S.M., Cummings J.H. (1992). Classification and measurement of nutritionally important starch fractions. Eur. J. Clin. Nutr..

[6] Bodinham C.L., Smith L., Thomas E.L., Bell J.D., Swann J.R., Costabile A., Russell-Jones D., Umpleby A.M., Robertson M.D. (2014). Efficacy of increased resistant starch consumption in human type 2 diabetes. Endocr. Connect..

[7] Nichenametla S.N., Weidauer L.A., Wey H.E., Beare T.M., Specker B.L., Dey M. (2014). Resistant starch type 4-enriched diet lowered blood cholesterols and improved body composition in a double blind controlled cross-over intervention. Mol. Nutr. Food Res..

[8] Zhang Y., Wang Y., Zheng B., Lu X., Zhuang W. (2013). The in vitro effects of retrograded starch (resistant starch type 3) from lotus seed starch on the proliferation of Bifidobacterium adolescentis. Food Funct..

[9] Gibson G.R., Probert H.M., van Loo J., Rastall R.A., Roberfroid M.B. (2004). Dietary modulation of the human colonic microbiota: Updating the concept of prebiotics. Nutr. Res. Rev..

[10] Sun Q., Dai L., Nan C., Xiong L. (2014). Effect of heat moisture treatment on physicochemical and morphological properties of wheat starch and xylitol mixture. Food Chem..

[11] Morales-Medina R., del Mar Munio M., Guadix E.M., Guadix A. (2014). Production of resistant starch by enzymatic debranching in legume flours. Carbohydr. Polym..

[12] Shin S.I., Lee C.J., Kim D.-I., Lee H.A., Cheong J.-J., Chung K.M., Baik M.-Y., Park C.S., Kim C.H., Moon T.W. (2007). Formation, characterization, and glucose response in mice to rice starch with low digestibility produced by citric acid treatment. J. Cereal Sci..

[13] Aparicio-Saguilan A., Gutierrez-Meraz F., Garcia-Suarez F.J., Tovar J., Bello-Perez L.A. (2008). Physicochemical and functional properties of cross-linked banana resistant starch. Effect of pressure cooking. Starch Starke.

[14] Guo J., Liu L., Lian X., Li L., Wu H. (2014). The properties of different cultivars of Jinhai sweet potato starches in China. Int. J. Biol. Macromol..

[15] Tian S.J., Rickard J.E., Blanshard J.M.V. (1991). Physicochemical properties of sweet potato starch. J. Sci. Food Agric..

[16] Abegunde O.K., Mu T.-H., Chen J.-W., Deng F.-M. (2013). Physicochemical characterization of sweet potato starches popularly used in Chinese starch industry. Food Hydrocoll..

[17] Reddy C.K., Suriya M., Haripriya S. (2013). Physico-chemical and functional properties of resistant starch prepared from red kidney beans (*Phaseolus vulgaris* L.) starch by enzymatic method. Carbohydr. Polym..

[18] Zhang Y., Zeng H., Wang Y., Zeng S., Zheng B. (2014). Structural characteristics and crystalline properties of lotus seed resistant starch and its prebiotic effects. Food Chem..

[19] Gerits L.R., Pareyt B., Delcour J.A. (2015). Wheat starch swelling, gelatinization and pasting: Effects of enzymatic modification of wheat endogenous lipids. LWT Food Sci. Technol..

[20] Hung P.V., Vien N.L., Lan Phi N.T. (2016). Resistant starch improvement of rice starches under a combination of acid and heat-moisture treatments. Food Chem..

[21] Sun Q., Han Z., Wang L., Xiong L. (2014). Physicochemical differences between sorghum starch and sorghum flour modified by heat-moisture treatment. Food Chem..

[22] Kaur A., Singh N., Ezekiel R., Guraya H.S. (2007). Physicochemical, thermal and pasting properties of starches separated from different potato cultivars grown at different locations. Food Chem..

[23] Gomes A.M.M., Silva C.E.M.D., Ricardo N.M.P.S. (2005). Effects of annealing on the physicochemical properties of fermented cassava starch (*Polvilho azedo*). Carbohydr. Polym..

[24] Vermeylen R., Goderis B., Delcour J.A. (2006). An X-ray study of hydrothermally treated potato starch. Carbohydr. Polym..

[25] Singh N., Singh J., Kaur L., Singh Sodhi N., Singh Gill B. (2003). Morphological, thermal and rheological properties of starches from different botanical sources. Food Chem..

[26] Cheetham N.W.H., Tao L. (1998). Variation in crystalline type with amylose content in maize starch granules: An X-ray powder diffraction study. Carbohydr. Polym..

[27] Zobel H.F. (1988). Molecules to Granules: A Comprehensive Starch Review. Starch Starke.

[28] Huang T.T., Zhou D.N., Jin Z.Y., Xu X.M., Chen H.Q. (2015). Effect of debranching and heat-moisture treatments on structural characteristics and digestibility of sweet potato starch. Food Chem..

[29] Zeng S., Wu X., Lin S., Zeng H., Lu X., Zhang Y., Zheng B. (2015). Structural characteristics and physicochemical properties of lotus seed resistant starch prepared by different methods. Food Chem..

[30] Diop C.I., Li H.L., Xie B.J., Shi J. (2011). Effects of acetic acid/acetic anhydride ratios on the properties of corn starch acetates. Food Chem..

[31] Flores-Morales A., Jiménez-Estrada M., Mora-Escobedo R. (2012). Determination of the structural changes by FT-IR, Raman, and CP/MAS 13C NMR spectroscopy on retrograded starch of maize tortillas. Carbohydr. Polym..

[32] Joshi M., Aldred P., McKnight S., Panozzo J.F., Kasapis S., Adhikari R., Adhikari B. (2013). Physicochemical and functional characteristics of lentil starch. Carbohydr. Polym..

[33] Zhang J., Chen F., Liu F., Wang Z.-W. (2010). Study on structural changes of microwave heat-moisture treated resistant Canna edulis Ker starch during digestion in vitro. Food Hydrocoll..

[34] Wei C., Qin F., Zhou W., Xu B., Chen C., Chen Y., Wang Y., Gu M., Liu Q. (2011). Comparison of the crystalline properties and structural changes of starches from high-amylose transgenic rice and its wild type during heating. Food Chem..

[35] Van Soest J.J.G., Tournois H., de Wit D., Vliegenthart J.F.G. (1995). Short-range structure in (partially) crystalline potato starch determined with attenuated total reflectance Fourier-transform IR spectroscopy. Carbohydr. Res..

[36] Sevenou O., Hill S.E., Farhat I.A., Mitchell J.R. (2002). Organisation of the external region of the starch granule as determined by infrared spectroscopy. Int. J. Biol. Macromol..

[37] Bird A.R., Vuaran M., Crittenden R., Hayakawa T., Playne M.J., Brown I.L., Topping D.L. (2009). Comparative effects of a high-amylose starch and a fructooligosaccharide on fecal bifidobacteria numbers and short-chain fatty acids in pigs fed Bifidobacterium animalis. Dig. Dis. Sci..

[38] Miao M., Jiang H., Jiang B., Cui S.W., Jin Z., Zhang T. (2012). Structure and functional properties of starches from Chinese ginkgo (*Ginkgo biloba* L.) nuts. Food Res. Int..

[39] Wang L., Xie B., Shi J., Xue S., Deng Q., Wei Y., Tian B. (2010). Physicochemical properties and structure of starches from Chinese rice cultivars. Food Hydrocoll..

